# Proteolytic cleavage analysis at the Murray Valley encephalitis virus NS1-2A junction

**DOI:** 10.1186/s12985-015-0375-4

**Published:** 2015-09-17

**Authors:** Siti Nor Khadijah Addis, Eva Lee, Jayaram Bettadapura, Mario Lobigs

**Affiliations:** John Curtin School of Medical Research, The Australian National University, Canberra, ACT Australia; School of Fundamental Science, Universiti Malaysia Terengganu, 21030 Kuala Terengganu, Terengganu Malaysia; Faculty of Education, Science, Technology and Mathematics, University of Canberra, Canberra, ACT Australia; Emerging Viruses & Inflammation Research Group, Institute for Glycomics, Griffith University, Nathan, QLD Australia; Australian Infectious Diseases Research Centre, School of Chemistry and Molecular Biosciences, The University of Queensland, St Lucia, QLD Australia

## Abstract

**Background:**

Our understanding of the proteolytic processing events at the NS1-2A junction in the flavivirus polyprotein has not markedly progressed since the early work conducted on dengue virus (DENV). This work identified an octapeptide sequence located immediately upstream of the cleavage site thought to be important in substrate recognition by an as yet unknown, endoplasmic reticulum-resident host protease. Of the eight amino acid recognition sequence, the highly conserved residues at positions P1, P3, P5, P7 and P8 (with respect to N-terminus of NS2A) are particularly sensitive to amino acid substitutions in terms of DENV NS1-NS2A cleavage efficiency; however, the role of the octapeptide in efficient NS1 and NS2A production of other flaviviruses has not been experimentally addressed.

**Methods and Results:**

Using site-directed mutagenesis at the NS1-2A cleavage site of Murray Valley encephalitis virus (MVEV), we confirmed the dominant role of conserved octapeptide residues for efficient NS1-2A cleavage, while changes at variable and the P1’ residues were mostly tolerated. However, digressions from the consensus cleavage motif derived from studies on DENV were also found. Thus, comparison of the impact on cleavage of mutations at the NS1-2A junction of MVEV and DENV showed virus-specific differences at both conserved and variable residues.

**Conclusion:**

We show, with subgenomic expression and infectious clone-derived mutants of MVEV that conserved residues in the flavivirus octapeptide motif can be replaced with a different amino acid without markedly reducing cleavage efficiency of NS1 and NS2A.

## Introduction

Flavivirus gene expression involves translation of the genomic RNA (~11 kb) into a single polyprotein, which traverses the membrane of the endoplasmic reticulum (ER) multiple times. Proteolytic processing of this polyprotein into 3 structural and at least 7 non-structural (NS) viral gene products is catalysed, with the exception of one cleavage, by two enzymes: the virally encoded NS2B-3 protease located in the cytosol and the endoplasmic reticulum (ER) luminal host enzyme, signal peptidase (reviewed in reference [1, 2]). The identity of the protease, which cleaves at the junction of the NS1 and NS2A proteins remains elusive.

Our understanding of the NS1-2A cleavage event has not progressed since the early work conducted on Dengue virus (DENV), which defined the cleavage site and essential determinants for efficient processing [3–6]. The cleavage consensus sequence is comprised of 8 amino acids at the C-terminus of NS1 and displays striking amino acid sequence conservation among the flaviviruses at residues, P1, P3, P5, P7 and P8 (relative to the cleavage site). However, high amino acid variability is present at residues P2 and P4, while P6 mostly accommodates a basic amino acid or a Gln; moreover, and the first amino acid down-stream of the cleavage site (P1’) is also highly variable between the flaviviruses. This ‘octapeptide motif’ is the minimal sequence requirement in NS1 for cleavage at the NS1-2A junction, given that deletion of all other NS1 sequence, except the N-terminal signal peptide, allowed proteolytic processing to occur [3–5]. The signal peptide is required for translocation of NS1 and thereby ascertains ER luminal location of the NS1-2A junction, from which was concluded that the protease which catalyses the cleavage also resides in this intracellular compartment [3].

Previous research on proteolytic processing at the flavivirus NS1-2A junction has been restricted exclusively to subgenomic expression models of the corresponding polyprotein region of DENV. While the generality to other flaviviruses of the findings for DENV has not yet been confirmed, it is also unclear whether insights gained from subgenomic expression experiments apply to viral infections. It is also intriguing to determine whether the pattern of absolute amino acid sequence conservation in the octapeptide is required exclusively for recognition of the NS1-2A cleavage site by the protease, or whether it serves an additional function in replication. Here, we have addressed these questions by mutational analysis of the NS1-2A cleavage site in recombinant expression and mutant virus infections using Murray Valley encephalitis virus (MVEV) as a model.

## Results

### Mutational analysis of the MVEV NS1-2A cleavage site

**Fig. 1:**
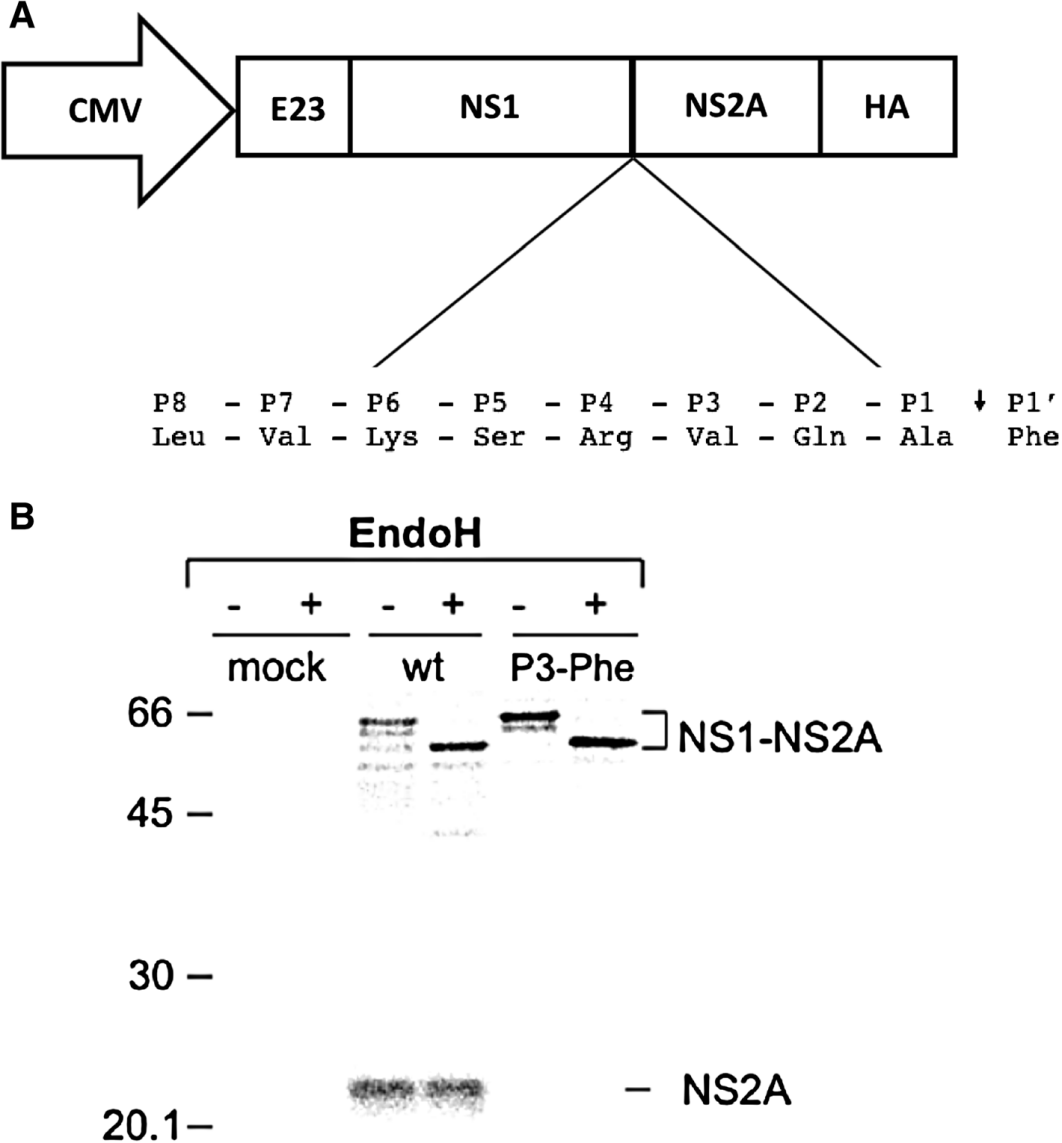
Design and expression of plasmid expressing *ns1* and *ns2A* gene. **a** The pRc/CMV.NS1-2A.HA plasmid contain an N-terminal signal sequence consisting of the last 23 residue of MVEV E protein and encodes an HA tag at the C terminus of NS2A. Shown below is the amino acid sequence of the MVEV octapeptide (P1 – P8) with the N-terminal residue of NS2A (P1’). **b** Cleavage at the NS1-2A junction of wt and octapeptide mutant construct, P3-Phe. Lysates of radiolabelled COS-7 cells transfected with wt or mutant construct, P3- Phe, or mock-transfected, were immunoprecipitated with an anti-HA-tag mAb. The recovered proteins were treated with endoH (+) or left untreated (−), and analysed by SDS- PAGE (12.5 % acrylamide). Position and size (in kilodaltons) of marker proteins are indicated on left. Bands corresponding to cleaved NS2A, and glycosylated and deglycosylated NS1-2A precursors are labelled. The data are representative of three independent experiments

An NS1-2A eukaryotic expression cassette containing the authentic signal peptide of NS1 plus an HA-tag, C- terminally fused to the NS2A protein (to facilitate NS2A detections since no antibody against NS2A is currently available), was constructed for the mutational analysis of the NS1-2A cleavage site in the MVEV polyprotein (Fig. 1a). Following transient transfection of this DNA into COS-7 cells, the tagged NS2A protein (~24 kDa) and uncleaved NS1-2A precursor (64 – 66 kDa) were immunoprecipitated from cell lysates with a HA-specific mAb. A mutant construct (P3-Phe), in which NS1-2A cleavage was mostly abolished due to a Val to Phe substitution at the P3 residue in the octapeptide (see below), did not generate detectable NS2A, but NS1-2A precursor bands of increased intensity relative to wild-type (wt) were observed. Analysis of N-linked glycosylation by endoH digestion showed sensitivity of two of three putative precursor bands to the deglycosylation treatment and a shift in electrophoretic mobility to that of the faster migrating form (Fig. 1b). This is consistent with the presence of three N-linked glycans on the MVEV NS1 protein, of which two moieties are sensitive to endoH digestion [7], and suggests that these protein bands represent glycosylation variations of the NS1-2A precursor protein. The effective recovery by immunoprecipitation of the precursor and the NS2A cleavage product implies that recombinant expression of the NS1-2A polyprotein fragment followed by HA-tag-specific immunoprecipitation of NS2A and related products allows estimation of cleavage efficiency at the NS1-2A junction, as described in the Materials and Methods. Immunoprecipitation with MVEV NS1-specific mAbs [8] resulted in recovery of the NS1 protein from lysates of cells transfected with the NS1-2A expression plasmid. However, the antibodies failed to react with the NS1-2A precursor, even when a construct with poor cleavage efficiency at the NS1-2A junction was used and for which the production of the precursor was verified by parallel immunoprecipitation with the HA- specific antibody. This prevented the use of available NS1-specific antibodies for calculation of cleavage efficiency at the NS1-2A junction, although comparison of NS1 band intensities corresponding to constructs with different cleavage efficiencies at the junction corroborated results obtained from NS2A-specific immunoprecipitations (data not shown).

**Fig. 2:**
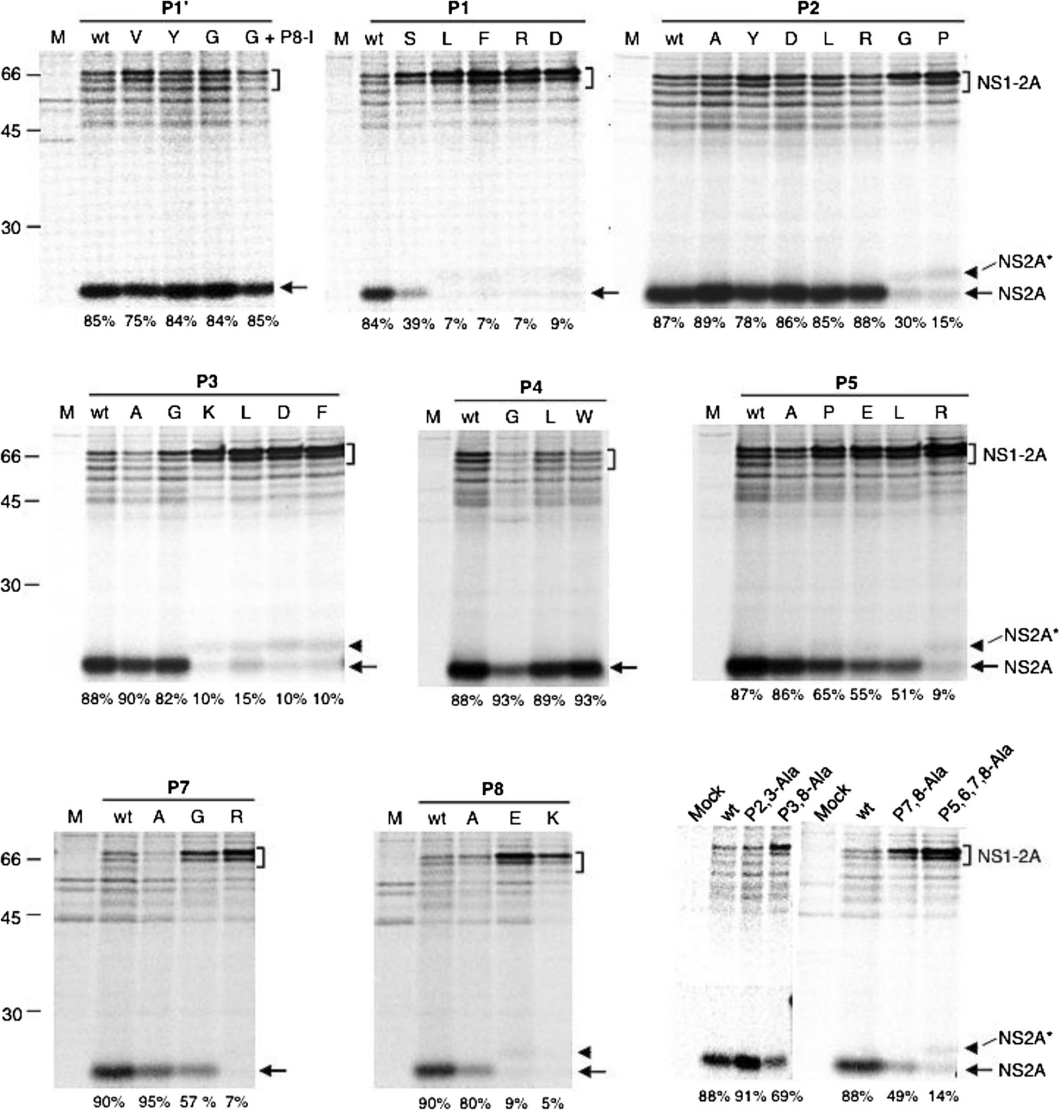
Mutational analysis of the MVEV NS1-2A cleavage site. COS-7 cells were transfected with pNS1-2A.HA wt plasmid DNA or mutant constructs with amino acid substitutions in the octapeptide motif or at the P1’ residue. At 2 days post-transfection, the cells were metabolically labelled for 0.5 h and chased for 0.5 h. Cell lysates were subjected to immunoprecipitation with an anti-HA tag mAb and proteins separated by SDS-PAGE (12.5 % acrylamide). Mutations introduced at the NS1-2A cleavage site are indicated at the top of each panel and % cleavage is shown under each lane. Position and size (in kilodaltons) of marker proteins are indicated on left, and bands corresponding to NS1-2A precursors and cleaved NS2A are denoted by a bracket and arrow, respectively. Arrowheads indicate a putative NS2A related product (NS2A*) of larger size than authentic NS2A. Data are representative of at least 3 independent experiments

Figure 2 shows the representative data on the effect of mutations at the MVEV NS1-2A cleavage site on proteolytic processing of NS1 and NS2A; cleavage efficiencies for wt and mutant constructs are summarized in Table 1.

**Table 1:** Effect of mutations introduced into the MVEV octapeptide and at the P1’ residue on NS1-2A cleavage

P8	P7	P6	P5	P4	P3	P2	P1	P1’	% cleavage^a^ (mean ± SEM)
L	V	K	S	R	V	Q	A	F	88 ± 1
								Y	84 ± 0
								V	80 ± 5
								G	86 ± 3
							S		53 ± 2
							L		6 ± 1
							F		7 ± 0
							R		7 ± 0
							D		10 ± 1
						A			88 ± 2
						Y			79 ± 3
						G			37 ± 4
						D			89 ± 4
						L			89 ± 2
						P			11 ± 4
						R			87 ± 1
					A				88 ± 2
					L				23 ± 3
					G				83 ± 1
					K				14 ± 2
					D				10 ± 0
					F				7 ± 3
				E					91 ± 4
				G					91 ± 3
				L					83 ± 5
				W					92 ± 1
			A						83 ± 2
			R						13 ± 4
			L						53 ± 2
			E						57 ± 2
			P						67 ± 2
	A								94 ± 2
	G								60 ± 3
	R								11 ± 4
A									80 ± 3
E									9 ± 0
K									8 ± 3
I								G	91 ± 2
					A	A			91 ± 2
A					A				69 ± 5
A	A								60 ± 5
A	A	A	A						28 ± 4

^*a*^Percent NS1-2A cleavage was calculated as described in the Materials and Methods; mean values of at least 3 independent experiments ± the standard error of the mean (SEM) are given

#### Substitutions at P1’

The first amino acid down-stream of the NS1-2A cleavage site is not conserved among the flaviviruses. Substitution of Phe at the P1’ position in MVEV with a aromatic residue (Tyr), or the non-conservative changes to Gly or Val, did not markedly impact on cleavage efficiency, indicating that amino acid side chain variability is tolerated at this position. A serendipitous change at P8 in the octapeptide (Leu→Ile) in a mutant construct harbouring the P1’ Gly substitution also had no marked effect on cleavage efficiency relative to wt.

#### Substitutions at P1

Changes at this conserved octapeptide residue dramatically reduced cleavage of NS1 and NS2A. While a conservative Ala→Ser substitution resulted in partial cleavage (52 %), the non-conservative changes to Leu, Phe, Arg or Asp were not tolerated (<10 % cleavage).

#### Substitutions at P2 and P4

Non-conservative changes at the P2 and P4 octapeptide residues had, in most instances, no effect on cleavage efficiency, consistent with the high variability at these residues among the flaviviruses. Nevertheless, two substitutions were identified, which significantly reduced cleavage: replacement of P2 Gln with Gly or Pro resulted in only 30 and 15 % cleavage, respectively. Accordingly, the introduction at P2 of amino acids that significantly affect the conformational flexibility of the protein backbone, but not those that change the electrostatic or steric properties of the side chain, was detrimental to cleavage site recognition by the protease.

An additional band with a mass of ~23.5 kDa (NS2A*) was frequently observed for constructs with poor cleavage of NS1 and NS2A (Fig. 2). Given the larger size than HA-tagged NS2A, it is possible that cleavage up-steam of the NS1-2A junction may occur when processing at the authentic cleavage site is prevented due to the introduction of mutations into the octapeptide.

#### Substitutions at P3

Substitution of Val, which is invariant among all flaviviruses, with charged (Lys and Asp) or bulky and hydrophobic amino acids (Leu and Phe) produced predominantly the uncleaved NS1-2A precursor. Surprisingly, the replacement of Val with small, aliphatic amino acids (Gly and Ala) was well tolerated. This shows, for the first time, that the strictly conserved P3 octapeptide residue can be substituted with a different amino acid without markedly reducing NS1-2A cleavage in a subgenomic expression system.

#### Substitutions at P5

Cleavage at the MVEV NS1-2A junction showed a partial tolerance to the effect of introduction of non-conservative substitutions at the conserved P5 octapeptide residue. While the replacement of P5 Ser with a basic amino acid (Arg) abolished cleavage, that with an acidic (Glu), hydrophobic (Leu) or strong helix-breaking residue (Pro) reduced cleavage efficiencies to ~50 – 60 %. Similar to our observation for the P3 residue, the introduction of Ala at P5 did not impact on cleavage efficiency relative to wt.

#### Substitutions at P7 and P8

A small number of changes were tested at the N-terminal two conserved octapeptide residues. For both residues, it was found that Ala mutations did not affect cleavage efficiency, while the introduction of a charged residue prevented NS1-2A processing. A Val→Gly change at P7 resulted in only partially inhibition of cleavage.

#### Effect of multiple Ala substitutions

Mutagenesis of single residues in the MVEV octapeptide showed that the introduction of Ala at each of the conserved positions did not markedly affect cleavage of NS1-2A (Table 1). To test whether there was a synergistic effect on cleavage of multiple Ala mutations in the octapeptide, constructs with Ala substitutions at two or four residues were produced. Simultaneous Ala mutations at the non-conserved P2 and conserved P3 positions (P2, 3-Ala) did not significantly alter cleavage efficiency relative to wt. In contrast, Ala substitutions at two conserved positions (P3, 8-Ala and P7, 8-Ala) resulted in partial (60 – 70 %) NS1-2A cleavage and a mutant construct with Ala substitutions from P5 through to P8 (P5, 6, 7, 8-Ala) showed poor (28 %) cleavage (Table 1). Therefore, it appears multiple Ala mutations have a synergistic, detrimental effect on NS1-2A cleavage.

### Recovery of NS1-2A cleavage site mutant viruses

**Fig. 3:**
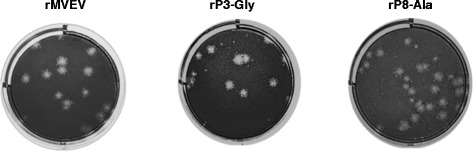
Plaque morphology of rMVEV and NS1-NS2A cleavage site mutant viruses. Samples of culture supernatant from BHK cells transfected with rMVEV, rP3-Gly or rP8-Ala were subjected to plaque assay on Vero cells. The corresponding plaque morphology on Vero cell monolayers stained with 0.02 % neutral red on day 4 p.i and recorded the following day are given

To investigate the importance of conserved residues at the NS1-2A cleavage site for virus growth, two octapeptide mutations were introduced independently into a full-length infectious clone of MVEV (Table 2). The changes made were based on the results from subgenomic expression of the NS1-2A polyprotein region, and included the non-conservative Val to Gly substitution at residue P3, which is occupied by an identical amino acid in all flaviviruses, and an Ala substitution at the highly conserved P8 position in the octapeptide [4]. Both NS1-2A cleavage site mutant constructs gave rise to viable virus following electroporation of full-length RNA into BHK cells, and there was no significant plaque size difference between the mutant viruses and wt (Table 2; Fig. 3). The genotype of the mutant viruses was stable in the E to NS2B polyprotein gene region following three passages in BHK cell culture.

**Table 2:** Recovery of MVEV NS1-2A cleavage site mutants

Virus	Octapeptide motif and mutation ^*a*^	Plaque size ^*b*^ (Fig. 3)	NS1-2A cleavage (from Table 1)
rMVEV	L-V-K-S-R-V-Q-A ↓	4 mm	88 %
rP3-Gly	L-V-K-S-R-G-Q-A ↓	4 mm	83 %
rP8-Ala	A-V-K-S-R-V-Q-A ↓	4 mm	80 %

^*a*^Mutations introduced into the octapeptide are in bold and underlined, and arrow denotes the NS1-2A cleavage site

^*b*^Plaque size on Vero cell monolayers stained with neutral red at 4 days pi and observed 16 h later

### Growth and RNA synthesis of NS1-2A cleavage site mutants

**Fig. 4:**
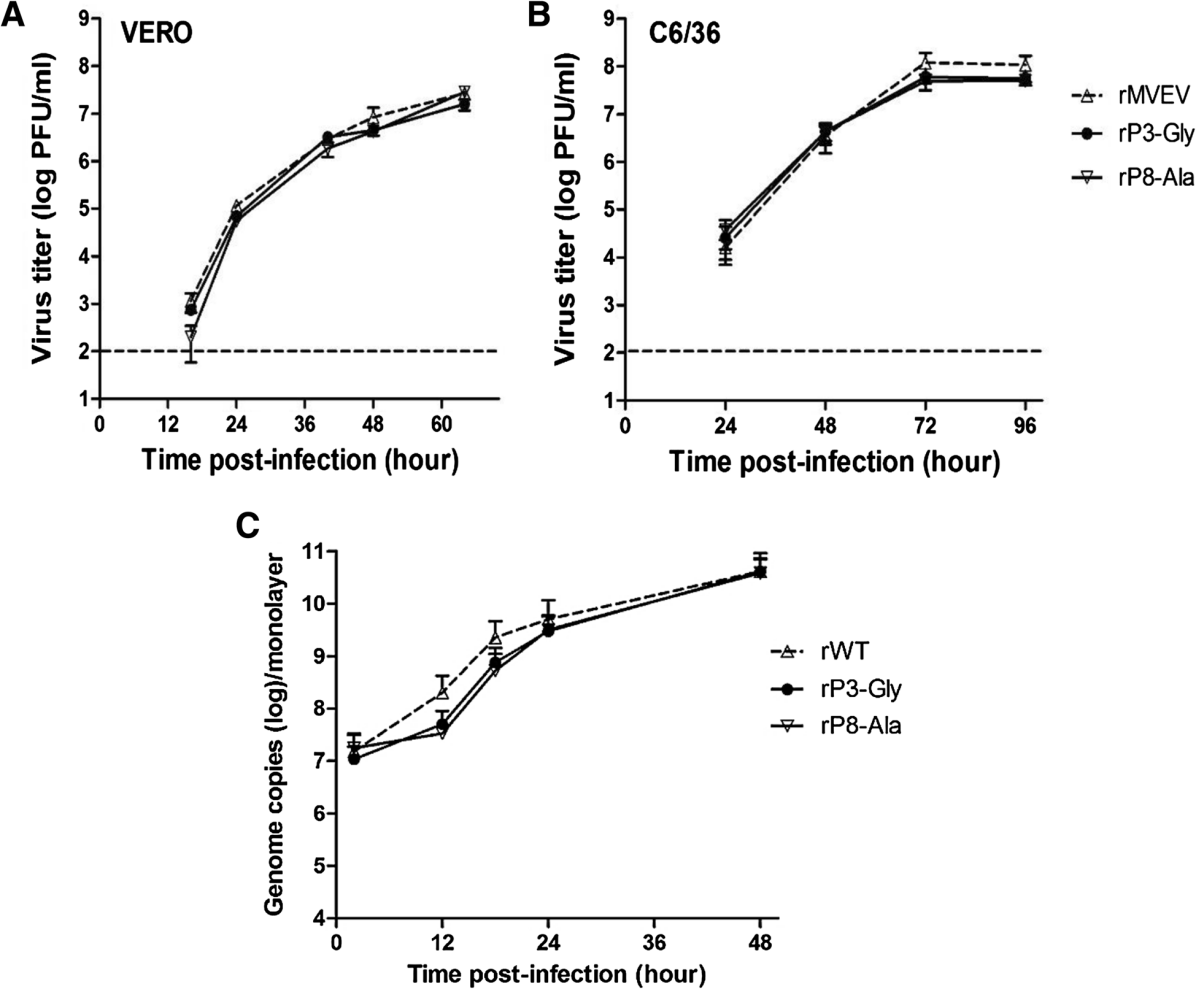
Growth and RNA synthesis analysis. **a** Growth of rMVEV and NS1-2A cleavage site mutants in mammalian and insect cells. Vero and C6/36 cells were infected at a multiplicity of ~0.1. Virus titers in growth samples were measured by plaque assay on Vero cells. The interrupted line denotes the detection limit of virus yield. Error bars indicate the standard error of the mean (SEM) of two independent experiments. **b** Virus-specific RNA in infected Vero cells. Vero cells were infected with rMVEV or NS1-2A cleavage site mutants (MOI ~ 1) and total intracellular RNA extracted at the indicated time points. Virus-specific RNA was measured by real-time qRT-PCR. Error bars indicate the SEM for two determinations for each sample

Growth of NS1-2A cleavage site mutants in mammalian (Vero) and mosquito (C6/36) cells was compared to that of an infectious clone-derived MVEV (rMVEV). Virus infections were performed at MOI ~0.1 and extracellular virus titers in growth samples were determined by plaque titration on Vero cells. Growth in Vero cells of rP3-Gly and rP8-Ala did not differ markedly (<2-fold) from that of wt during the 16 – 64 h time interval, except for a ~10-fold lower titer for rP8-Ala at 16 h pi (Fig. 4a). The wt rMVEV grew to high titers in C6/36 cells and reached a plateau of ~1 × 10^8^ PFU/ml at 72 h pi (Fig. 4b); mutants rP3-Gly and rP8-Ala showed a similar growth phenotype in terms of kinetics and maximum titers reached, with the latter only marginally lower (<2-fold). Accordingly, mutations at conserved octapeptide residues that allow efficient proteolytic processing at the NS1-2A junction in the subgenomic expression assay are minimally detrimental to virus growth in the Vero and C6/36 cells. Moreover, the result also indicates that NS1-2A cleavage efficiencies determined by subgenomic expression are representative of that in viral infection.

The NS1 and NS2A proteins function in RNA replication [9] and as constituents of the viral replication complex [10–12]. Hence, the production and intracellular accumulation of viral RNA was assessed in wt and mutant virus infected Vero cells by real-time qRT-PCR (Fig. 4c). Wild-type virus-specific RNA synthesis was apparent at 12 h pi, when the level of intracellular viral RNA had increased ~25-fold relative to cell-associated residual input. Over the following 12 h, intracellular viral RNA continued to increase exponentially and at 48 h pi an approximately three log increase relative to residual input was found. Variants rP3-Gly and rP8-Ala displayed a slower kinetics of intracellular RNA accumulation than wt but reached a comparable level at 48 h pi.

### Growth and virulence in mice

**Fig. 5:**
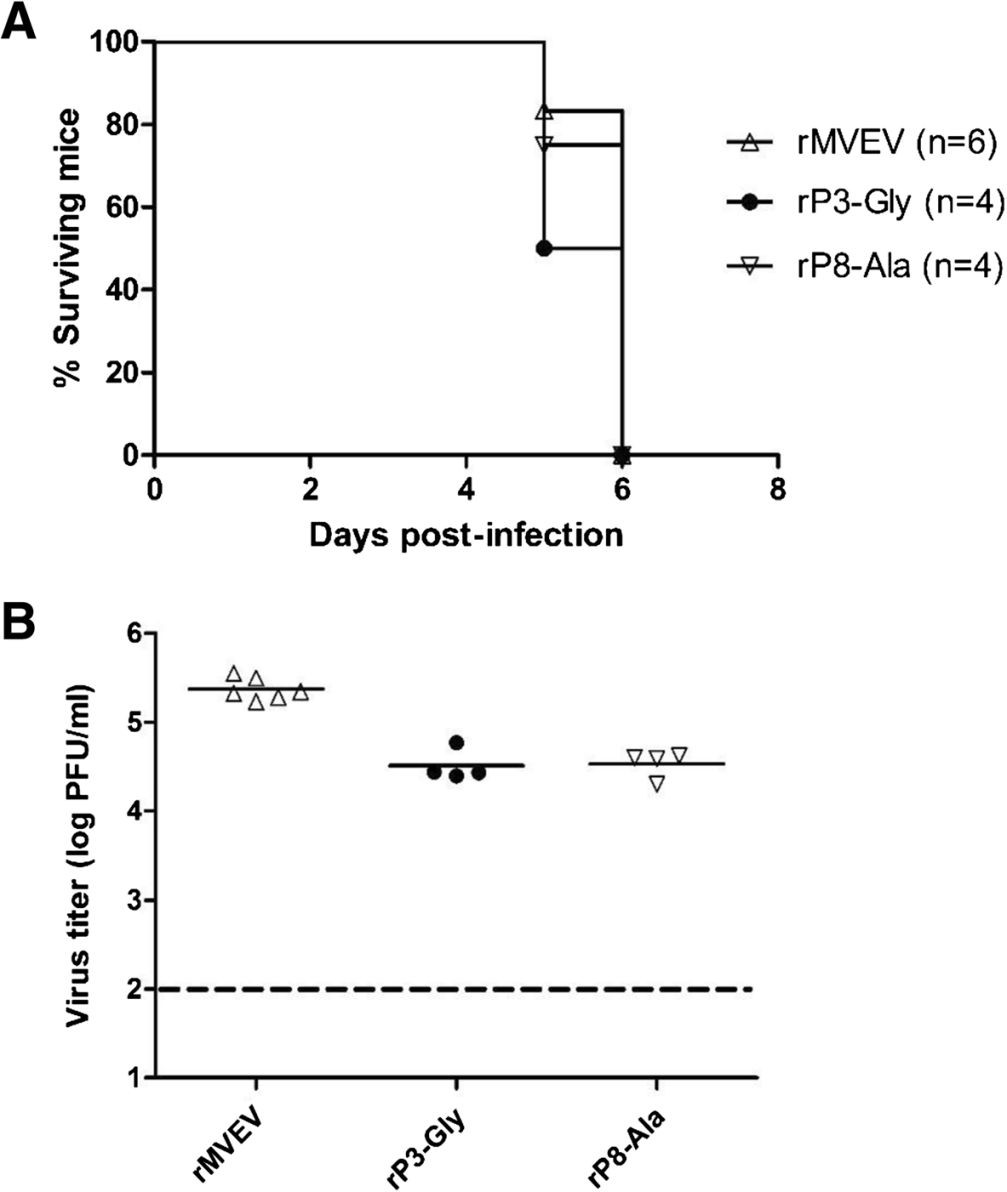
Virulence and growth in IFN-α-R−/− mice. Groups of 6-week-old mice were infected, i.p., with 10^3^ PFU of rMVEV, NS1-2A cleavage site mutants, or plaque size variants derived thereof, and morbidity and mortality recorded daily (**a**). At 2 days pi, serum was collected from infected mice and viremia titers determined by plaque assay on Vero cells (**b**). Each symbol represents an individual mouse, and mean titers are represented by a horizontal line. The interrupted line indicates the detection limit of the plaque assay

Mice deficient in the type I interferon (IFN) response (IFN-α-R−/− mice) are highly susceptible to infection with MVEV by an extraneural route [13]; infection of 6-week-old mice with a low dose of wt rMVEV results in uncontrolled virus growth, a high viremia on day 2 pi and uniform mortality by day 6 pi (Fig. 5a and b). Variants rP3-Gly and rP8-Ala produced an ~10-fold lower viremia (*P* = 0.01), but 100 % mortality with the same average survival time relative to wt.

## Discussion

This is the first study that investigates the effect of polyprotein processing efficiency at the flavivirus NS1-2A junction on virus replication and growth in cell culture, and which provides insight into commonalities and differences in sequence requirements at the NS1-2A junction of two flaviviruses (MVEV and DENV) for cleavage by an as yet unidentified protease. The key finding in this study is the viability and efficient growth of mutants with changes at conservative residues in the octapeptide, with no significant impact on growth observed in cell culture.

### The consensus cleavage motif at the MVEV NS1-2A junction

This investigation on MVEV confirms the dominant role of the strictly conserved residues in the octapeptide at the C-terminus of NS1 for efficient NS1-2A cleavage, given that substitutions at these positions almost always greatly reduced cleavage. In contrast, non-conservative substitutions were often tolerated at the P1’, P2 and P4 sites, which are occupied by amino acids that differ in hydrophobicity and molecular bulk between different flaviviruses. Accordingly, sequence conservation in the octapeptide is predictive of the impact of substitutions on cleavage efficiency, supporting the view that conservation of physical and chemical properties of residues at P1, P3, P5, P7 and P8 is required to accommodate the cleavage site in the substrate-binding pocket of the protease.

Despite overall agreement with the ‘octapeptide rule’, several deviations were noted for MVEV. A non-conservative Val→Gly change at P3 did not markedly reduce efficiency of cleavage of NS1 and NS2A. In contrast, the conservative substitution of Val with Leu, a slightly larger aliphatic amino acid, was not tolerated. This suggests that small size of the side chain is the overriding property required at P3 in the cleavage sequence motif of MVEV; this proposition is supported by a second substitution of Val at P3 with a small side chain amino acid (Ala), which showed wt cleavage efficiency.

Of the other 21 changes at conserved octapeptide residues tested, only Ala substitutions at P5, P7 and P8, and a Leu to Ile change at P8 did not markedly affect NS1-2A cleavage. While replacement of Leu with Ile is a conservative change, the Ser→Ala change at P5 results in loss of polarity of the side chain and introduction of Ala for Val or Leu at P7 and P8, respectively, reduces side chain hydrophobicity and size. However, although eliminating the side chain beyond the β carbon, Ala substitutions do not alter the main-chain conformation nor do they impose extreme electrostatic or steric effects [14], which is the likely explanation why these changes had minimal impact on cleavage efficiency. On the other hand, the combination of two Ala substitutions at P7 and P8 or at P3 and P8 showed a reduction in cleavage to 60 and 68 %, respectively, and a further increase in the number of substitutions with Ala in the cleavage consensus sequence from P5 to P8 gave rise to very poor cleavage. This synergistic inhibitory effect on cleavage of two or more changes of conserved residues in the octapeptide to Ala illustrates yet again the important contribution of each of these residues in ascertaining efficient NS1-2A cleavage by a process, which most likely involves their role in proper alignment of the octapeptide with key residues inside the substrate-binding pocket of the protease.

The effect of mutations at two variable positions (P2 and P4) in the octapeptide on NS1-2A cleavage was also investigated. P4 showed greater tolerance to non-conservative amino acid changes than P2. Of the 11 mutations at P2 and P4, only the replacement of Gln at P2 with either Gly or Pro, two strong helix-breaking residues, markedly reduced cleavage. This suggests that alterations that significantly affect the conformational flexibility of the protein backbone, but not changes in electrostatic and steric properties of the side chain at P2 are detrimental to cleavage site recognition by the protease. Finally, this study also investigated the contribution of the P1’ residue to cleavage of NS1 and NS2A. No stringent requirement for conservation of the physical and chemical property of the aromatic side chain of Phe, which occupies this position in MVEV, was found.

### Comparison of MVEV and DENV-4 octapeptides

The most striking difference between the two viruses was the differential impact of a Val→Gly substitution at P3, which almost completely prevented cleavage in the DENV-4 polyprotein but had no marked effect on the production of MVEV NS1 and NS2A (Table 2). Other differences between the two viruses were seen for P1 Ala→Ser and P7 Val→Gly substitutions, which resulted in partial cleavage inhibition for MVEV but were not tolerated in the DENV-4 octapeptide, and for a P5 Ser→Pro change, which enhanced DENV-4 NS1-2A cleavage but was partially inhibitory in the case of MVEV (Table 3). A further difference in sequence requirement for NS1-2A cleavage between MVEV and DENV-4 was found at the P1’ position: substitutions leading to major changes in hydrophobicity and molecular bulk were tolerated in MVEV but not DENV-4. For instance, the introduction of Val at P1’ only marginally reduced cleavage efficiency at the MVEV but prevented cleavage at the DENV-4 NS1-2A junction [6].

**Table 3:** Comparison of the effect of changes at conserved residues in the octapeptide of MVEV and DENV-4 on NS1-2A cleavage

Octapeptide residue	Substitution	Cleavage efficiency	
		MVEV^*a*^	DENV-4^*b*^
P1 Ala	Ser	53 ± 2	10 ± 3
	Leu	6 ± 1	4 ± 4
	Phe	7 ± 0	0
	Arg	7 ± 0	5 ± 5
	Asp	10 ± 1	3 ± 2
P3 Val	Leu	23 ± 3	4 ± 3
	Gly	83 ± 1	10 ± 4
	Lys	14 ± 2	1 ± 2
P5 Ser	Ala	83 ± 2	100 ± 11
	Arg	13 ± 4	8 ± 3
	Pro	67 ± 2	132 ± 4
P7 Val	Gly	60 ± 3	1 ± 1
	Arg	11 ± 4	1 ± 1
P8 Leu or Met	Glu	9 ± 0	2 ± 1
	Lys	8 ± 3	2 ± 3

^*a*^Data from Table 1

^*b*^Data from reference [6]

Taken together, the mutational analyses of MVEV and DENV-4 octapeptides showed a small number of striking virus-specific differences in sequence requirement for NS1-2A cleavage, which may be imposed by one or more of several factors: (i) sequence variation at non-conserved residues, P2 and P4 (the P6 residue in MVEV and DEN-4 is Lys, and for most other flaviviruses either Lys or Arg); (ii) the P8 residue, which differs between MVEV (Leu) and DENV-4 (Met); (iii) the P1’ residue (Phe in MVEV and Gly in DEN-4); (iv) putative NS2A ‘cofactor-like’ activity (where the NH_2_-terminal ~ two-third of the NS2A protein was also indispensable for NS1-2A cleavage), given the significant amino acid sequence variation (22 %) in this protein between the two viruses.

### Effect of mutations at the NS1-NS2A cleavage site on virus viability, growth properties in cell culture and virulence in mice

Two changes in the octapeptide were introduced independently into a full-length infectious clone of MVEV. Since each construct gave a viable virus, we were able to undertake, for the first time, a comprehensive investigation on the role of NS1-NS2A cleavage in flavivirus replication. One of the main findings was the excellent correlation between NS1-NS2A cleavage efficiency in transient expression experiments and virus growth in mammalian and insect cells, notably when highly conserved octapeptide residues were substituted. Thus, variants rP3-Gly and rP8-Ala, which had mutations that only slightly reduced NS1-NS2A cleavage efficiency relative to wt, produced plaques on Vero cell monolayers of similar size to wt and grew in Vero and C6/36 cells with a kinetics that differed <2-fold from that of rMVEV.

A second major finding from this investigation was the tolerance of growth in cell culture of MVEV to the non-conservative Val→Gly change at P3 in the octapeptide, despite absolute conservation of Val at this position in the octapeptide among all flaviviruses. Similarly, an Ala substitution at the highly conserved P8 position in the octapeptide (either Leu or Met among all flaviviruses) did not markedly impact on virus macromolecular synthesis or growth in cell culture. This result shows that octapeptide mutations, which did not reduce cleavage at the NS1-NS2A junction in recombinant expression of the NS1-NS2A polyprotein region, also did not markedly impinge on RNA and virus replication in cell culture, regardless of amino acid conservation. From this we can conclude that the dominant role of the amino acid sequence conservation at the flavivirus NS1-NS2A cleavage site is in substrate recognition by the protease, which cleaves NS1 and NS2A. However, the question arises of why natural flavivirus isolates with comparable changes at conserved positions in the octapeptide as described here cannot be found. The likely explanation for this apparent contradiction is that Vero and C6/36 cells are relatively insensitive to mutations in the MVEV genome that are only marginally deleterious for virus replication, given that these cell culture systems are used because of a high virus yield, which may allow a significant level of wastage of viral gene products. This may not be the case in hosts and vectors essential for natural transmission of flaviviruses. In support of this proposition, we found that viremia in mice infected with variants rP3-Gly and rP8-Ala was significantly lower than in mice infected with wt rMVEV. A greater sensitivity of mouse relative to cell culture models in terms of impact of mutations on virus growth has also been noted for a MVEV variant with a mutation the C-prM junction in the flavivirus polyprotein [15].

## Conclusions

In summary, the comparative analysis between MVEV and DENV suggests that the octapeptide motif functions as a module rather than a specific determinant for NS1-NS2A cleavage that can accommodate virus-specific differences in the octapeptide region. Additional work is needed to define the precise mechanism of NS1-2A cleavage, which should ultimately lead to the identification of the protease responsible for this process and fill an important gap in the fundamental knowledge on the biology of flavivirus.

## Methods

### Cells

African green monkey kidney (Vero and COS-7) and baby hamster kidney (BHK-21) cells were cultured in Eagle’s minimum essential medium (MEM; Invitrogen) supplemented with 5 % heat-inactivated fetal calf serum (FCS), 0.1 mM non-essential amino acids (Invitrogen) and 100 U/ml penicillin-streptomycin (PSN; Invitrogen), and were grown in a humidified 37 °C CO_2_ incubator_._*Aedes albopictus* (C6/36) cells were maintained at 28 °C in MEM containing 10 % FCS, 0.1 mM non-essential amino acids and 100 U/ml PSN. All cells were from the American Type Culture Collection (ATCC).

### Eukaryotic expression plasmids

To generate plasmid pRc/CMV.NS1-2A.HA (7345 bp) encoding the MVEV NS1 and NS2A proteins (Fig. 1a), complementary deoxyribonucleic acid (cDNA) corresponding to the *ns1* and *ns2A* genes was cloned into pRc/CMV (Invitrogen) using *Hin*dIII/*Xba*I sites. The NS1 protein was preceded by its authentic signal peptide and the NS2A protein was C-terminally fused to an influenza virus hemagglutinin (HA) epitope tag (Tyr-Asp-Val-Pro-Asp-Tyr-Ala), which allows recovery of NS2A by immunoprecipitation with monoclonal antibody (mAb), 12CA5 [16]. To introduce amino acid substitutions in the octapeptide region, a fusion polymerase chain reaction (PCR) approach was employed [17] using the upstream primer, 5′-ACTGGATTGAGAGTGGACTCAATG-3′, and downstream primer, 5′-CTGATCAGCGAGCTCTAGCATTTAAGGTGA-3′, in combination with the corresponding mutagenesis primers (sequences will be provided upon request). Construction of mutant derivatives from plasmid, pRc/CMV.NS1-2A.HA, was by double digestion of mutagenized fragments (932 bp) and the corresponding *ns1-ns2A* gene region in the wt plasmid with restriction enzymes, *Ppu*MI and *Xba*I, and replacement of the wt with a mutated cDNA fragment. Ligation mixtures were transformed into *Escherichia coli* MC1061.1 cells, and the *ns1-ns2A* gene sequences verified by sequence analysis using BigDye v.3 (Applied Biosystems) at the Biomolecular Resources Facility in the John Curtin School of Medical Research in accordance with the manufacturer’s protocol.

### Transient expression in COS-7 cells, metabolic labelling, immunoprecipitation and sodium dodecyl sulphate polyacrylamide gel electrophoresis (SDS-PAGE)

Transfection of eukaryotic expression plasmid DNA into COS-7 cells was performed by the DEAE-dextran method, as described previously [18]. Metabolic labelling of proteins with Trans 35S-label (ICN), lysis with radio-immunoprecipitation assay (RIPA) buffer (50 m Tris–HCl, pH 7.4, 0.15 M NaCl, 1 % NP-40, 1 % sodium deoxycholate, 0.1 % SDS, 0.2 mM EDTA) containing a mammalian protease inhibitor cocktail (Biosciences), immunoprecipitation with 2 μl anti-HA epitope tag mAb (12CA5; 1.5 mg/ml), and SDS-PAGE were as described previously [19], with the variation of using a shorter labelling period (0.5 h followed by a 0.5 h chase) to prevent degradation of the NS2A protein. Following electrophoresis, gels were fixed by incubation for 30 min in 200 ml of 20 % acetic acid, thoroughly rinsed in distilled water and dried on Whatman 3 M paper using a gel dryer. Dried gels were placed in contact with a Photo-Imager screen (Fuji Film) for 3 to 4 days before the screen was scanned using a Fuji Film FLA/LAS or Typhoon FLA 9000 instrument. Image analysis for protein band quantitation was performed using Multi Gauge version 2.0 (Fuji Film).

For endoglycosidase H (endoH) treatment of immunoprecipitated proteins, bound immune complexes were eluted from the protein A-Sepharose beads by heating at 95 °C for 5 min in 30 μl of a denaturing buffer containing 1 % SDS, 10 mM Tris–HCl buffer (pH 6.8) and 5 % β-mercaptoethanol. The beads were removed by centrifugation and half of the supernatant was treated with an equal volume of digestion buffer containing 50 mM sodium citrate (pH 5.5) and 1 U of endoH (Roche) for 16 h at 37 °C. The other half of the supernatants received the same treatment in the absence of the enzyme.

### Estimation of NS1-2A cleavage efficiency

NS1-2A cleavage efficiency was calculated after normalising for the number of Met and Cys residues in uncleaved NS1-NS2A (30) and NS2A (14). The percentage cleavage efficiency was determined as the radioactivity (expressed as photostimulated luminescence, PSL) in the NS2A band divided by the sum of that in NS2A and the NS1-NS2A precursor bands using the formula: Cleavage (%) = [NS2A-specific PSL/(NS2A-specific PSL + NS1-2A precursor-specific PSL)] × 100.

### MVEV full-length infectious clone-derived viruses

To generate full-length cDNA clones of MVEV with specific mutation in the octapeptide motif, 776-bp *Nae*I-*Nhe*I fragments encompassing P3-Gly or P8-Ala mutations in NS1 were excised from the corresponding mutant pRc/CMV.NS1-2A.HA plasmids and ligated into a full-length MVEV cDNA clone, pMVEV-FL-v2 [20], replacing the wt *Nae*I-*Nhe*I fragment. Plasmid DNA (5 μg) was linearized by digestion with *Nsi*I, followed by blunt-ending with T4 DNA polymerase and in vitro transcription with T7 RNA polymerase, and RNA transcripts were electroporated into BHK-21 cells as described previously [20]. Culture medium was harvested after 3 days for virus titration by plaque assay on Vero cells as described previously [21], and frozen in aliquots at −70 °C. Sequence analysis of the entire E to NS2B polyprotein gene region was performed on plaque-purified virus stock. The genotypes of rP3-Gly and rP8-Ala viruses showed the introduced mutations and were stable following at least three passages in cell culture. The same observation was made for virus recovered from serum of infected mice.

### Viral RNA synthesis and accumulation in infected cells

Vero cells (3 × 10^5^ cells) in a 6-well tissue culture plate were infected with wt or mutant MVEV at multiplicity of infection (MOI) ~1. After 1 h of virus adsorption, the monolayers were washes twice with phosphate buffered saline (PBS) and 3 ml of growth medium was added, followed by incubation at 37°C. At 2, 12, 18, 24 and 48 h post-infection (pi), monolayers were washed twice with PBS, and total RNA was extracted using Trizol reagent^®^ (Invitrogen) and suspended in 20 μl of nuclease-free water. Quantitative real-time PCR (qRT-PCR) was performed as described previously [18].

### Animal experiments

All animal experiments were conducted in the animal handling facility operated by the Animal Breeding Establishment, John Curtin School of Medical Research (JCSMR) according to approved Australian National University (ANU) animal experimentation ethics standards for the care and use of laboratory animals. Virulence assays were performed in 3-week-old interferon-α receptor knock-out (IFN-α-R−/−) mice by intraperitoneal (i.p) inoculation with 10^3^ PFU of virus. Mice were monitored over a period of 28 days for the onset of disease and euthanized when signs of encephalitis were apparent. Blood was collected by tail bleeding at day 2 pi, and viremia levels were determined by plaque titration on Vero cells.

### Statistics

Differences in survival ratios in mouse challenge experiments were assessed using Mantel-Cox test. The Wilcoxon signed-rank test was applied to assess differences in viremia levels in infected IFN-α-R−/− mice for significance.
